# CD4/CD8 dual-positive mycosis fungoides: A case report and literature review

**DOI:** 10.1097/MD.0000000000022786

**Published:** 2020-10-16

**Authors:** Xiaojie Ding, Jia Chen, Le Kuai, Meng Xing, Yi Ru, Ying Luo, Yue Luo, Mi Zhou, Bin Li, Xin Li

**Affiliations:** a Department of Dermatology, Yueyang Hospital of Integrated Traditional Chinese and Western Medicine, Shanghai University of Traditional Chinese Medicine; b Department of Dermatopathology, Shanghai Dermatology Hospital, Tongji University; c Institute of Dermatology, Shanghai Academy of Traditional Chinese Medicine, Shanghai, China.

**Keywords:** case report, CD4/CD8, double positive, herbal medicine, mycosis fungoides

## Abstract

**Rationale:**

Mycosis fungoides (MF) is the most common cutaneous T-cell lymphoma. It appears as patches, plaques, and tumors depending on the stage of the disease, which presents a chronic progressive course. Compared to CD4^+^/CD8^-^ MF, CD4/CD8 dual-positive MF is an uncommon immune phenotype.

**Patient Concerns:**

A 36-year-old male patient presented with dryness and scales on his whole body.

**Diagnosis:**

The patient was diagnosed with MF based on results of pathological examination, immunohistochemical staining, and T-cell receptor gene rearrangement test.

**Interventions:**

The patient was advised to take an herbal medicine orally twice daily and apply a topical moisturizer after showering.

**Outcomes:**

After treatment and follow-up, the patient's symptoms of dryness and scales improved and his condition stabilized.

**Conclusions:**

While reviewing the literature, we found no previous reports on the treatment of dual-positive MF with Chinese medicine. In this report, we presented the first case of dual-positive MF successfully treated with Chinese medicine. The results suggest that oral ingestion of herbal medicine may be a feasible method for alleviating clinical symptoms of early stage MF. Therefore, the therapy should be explored for clinical use in the future.

## 1 Introduction

Mycosis fungoides (MF) is a primary cutaneous non-Hodgkin lymphoma of T-cell origin. It is relatively rare with an incidence of 5.6 per million persons.^[1]^ The etiology of MF is unclear and may be related to heredity, infection, and environment. MF can develop to involve the lymph nodes, peripheral blood, and viscera.^[2]^ The 3 typical cutaneous stages of MF are patches, infiltrated plaques, and tumors.^[3]^ Due to the nonspecific clinical manifestations, MF diagnosis is not easy to confirm in the early stages, resulting in improper medical treatment.^[4]^ The infiltrates of T-helper memory lymphocytes in MF lesions are generally found to be positive for CD3, CD4, and CD45RO and negative for CD8 phenotypes via immunohistochemical assays.^[5]^ In a preliminary, single-center prospective study, CD4 and CD8 co-expression characteristics were found in one-third of patients with MF through conventional immunophenotyping. These patients exhibited slightly lower rates of disease progression compared to patients with conventional CD4+/CD8− MF phenotype. These findings raise the possibility that co-expression of CD4 and CD8 in cutaneous lesions may confer a better prognosis in MF.^[6]^ Since the first CD4/CD8 dual-positive MF case described in 2014,^[7]^ only 5 cases (including ours) have been reported.^[8–10]^ Here, we present the case of a patient with CD4/CD8 dual-positive MF in accordance with the CARE guidelines,^[11]^ who responded well to treatment with Chinese medicine (CM).

## 2 Case presentation

A 36-year-old male engineer engaged in parts manufacturing and the decoration industry was referred to our hospital on September 12, 2012. He complained of dry and scaly skin across his entire body, which caused inconvenience in his daily life. He had been experiencing discomfort for 8 years without any obvious itching, burning, pain, or other discomfort. He denied any systemic symptoms, including fever, chills, nausea, vomiting, weight loss, night sweats, and abdominal pain. He denied a history of medical diseases such as diabetes, coronary heart disease, and infectious diseases. His vaccination status was unknown. He also denied any food and drug allergies. The patient reported no poor life habits and denied family history of hereditary disease. He applied moisturizer himself (details unknown) but the symptoms were not alleviated. He had been diagnosed with dermatitis, for which topical urea cream and oral vitamin C, vitamin E, and vitamin A were prescribed. However, his condition did not improve significantly. The patient had received intermittent treatment for 8 years and his condition had not been well controlled. In general, the disease condition was more severe in autumn/winter and milder in spring/summer.

Physical examination showed dry skin, diffused distribution of dark erythema, and various small white scales particularly on the neck, chest, and both upper limbs. There was mild erythema on the face and neck with unclear boundaries. The patient had a dry mouth, no sweat, and normal urine and bowel movements. The patient's tongue was red and covered with a thin and greasy coating, and pulse was fine and rapid.

**Figure 1:**
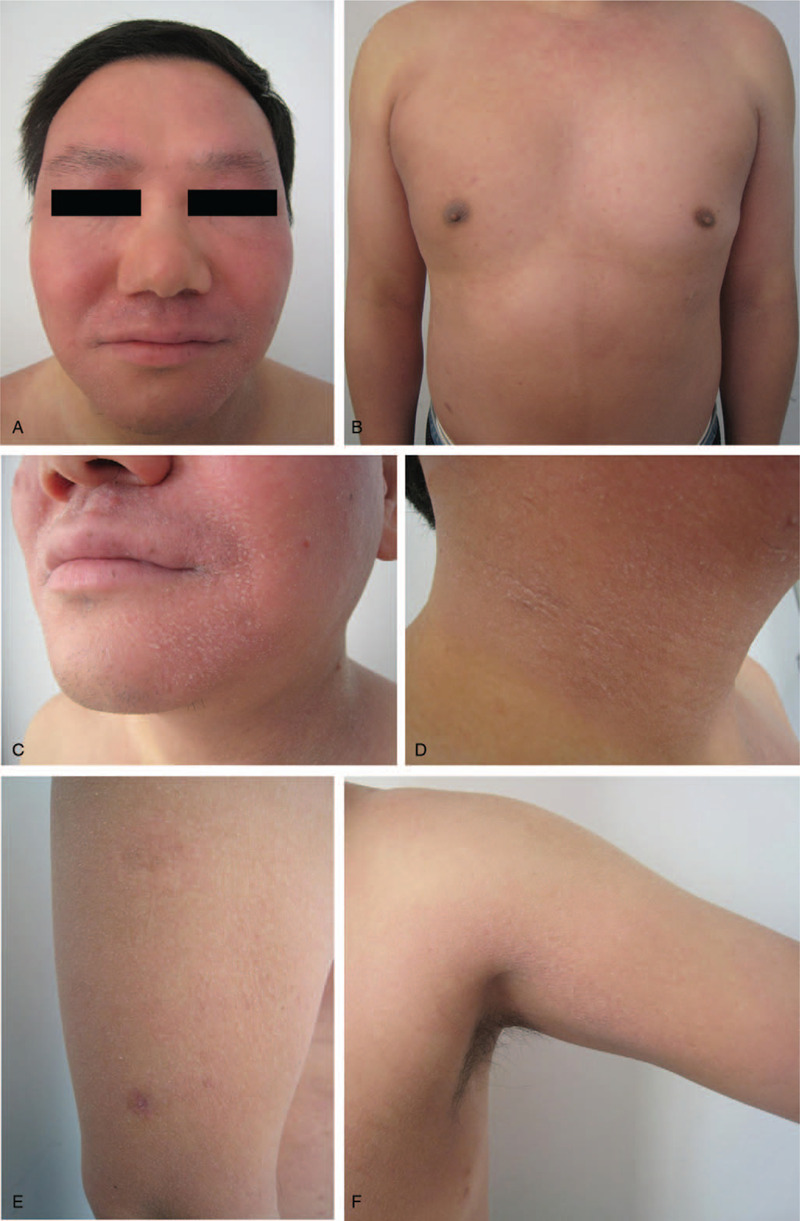
Clinical presentation of original symptoms. Distribution of dryness and scales on the face (A), chest (B), cheek (C), neck (D), and upper limbs (E–F). Dry skin, diffuse distribution of dark erythema, and some areas covered with small white scales.

Since the onset of the disease, the patient had no fever, chills, joint pain, swollen lymph nodes, weight loss, or history of trauma, foreign body contact, and suspicious drug use. The patient conveyed that he had undergone allergen tests, sex hormone tests, and tumor marker tests in the other hospital, but no abnormalities were detected. The patient's clinical symptoms of dry and scaly skin are shown in Figure 1.

Based on the clinical manifestations of dry and scaly skin, we could not easily make a clear diagnosis of the disease. The skin lesions of the patient were nonspecific and could be easily confused with diseases such as psoriasis and pityriasis rosea. To make a clear diagnosis, we took a biopsy of the skin from the arm and abdomen during examination, and the results were obtained within a week. Given the character of the skin lesions, we temporarily suspected vice psoriasis and CM diagnosis of “Yifeng Sores.” Tongue coating, pulse, and character of the skin lesions were consistent with blood-heat syndrome. We considered blood-heat and Yang floating as the main pathogenesis in CM theory. The patient was treated with herbal medicine orally for “cooling blood and restraining Yang” twice daily, 30 minutes after meals. Previous research conducted by our team showed that cooling blood and Yang restraining herbs had a positive clinical effect in the treatment of skin diseases such as blood-heat syndrome, eczema, and psoriasis vulgaris.^[12,13]^ In addition, experiments have shown that the drug for cooling blood and retraining Yang may function by upregulating the expression of PD-1 mRNA, PD-L2 mRNA, and their proteins in patients with psoriasis vulgaris, which is characterized by blood-heat syndrome.^[14]^ The specific composition, dosage, and medicinal parts of herbal components are shown in Table 1. In addition to oral herbal medicine, the patient was treated with a ceramide-linoleic acid -containing moisturizer (YuZe Skin Barrier Recovery Body Lotion, produced by Shanghai Jahwa United Company, China); the moisturizer was to be applied on the entire body after showering once daily.^[15]^

**Table 1:**
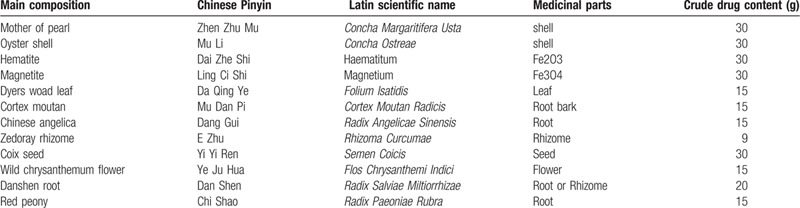
Standard formula of decoction of cooling blood and yang restraint recipe in English translation.

**Figure 2:**
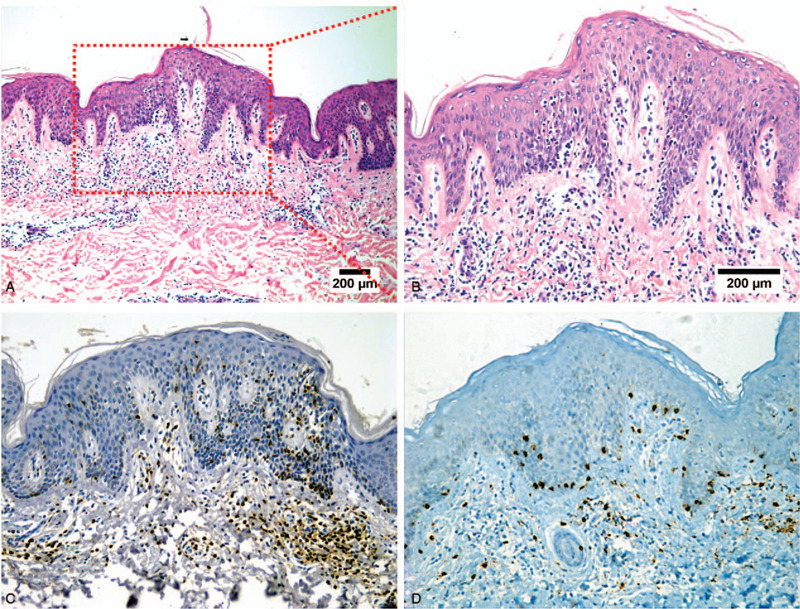
Histological findings and positive phenotype. (A, B) Microscopic examination revealed hyperkeratosis, parakeratosis, and mild acanthosis of the epidermis. Many atypical lymphocytes with medium-sized and hyperchromatic nuclei were observed in the epidermis. This epidermotropism consisted of lymphocytes colonizing the basal layer of the epidermis in a linear configuration. The dermal superficial band-like infiltrate was scant. (C) Neoplasm cells were positive for CD4 (200 ×). (D) Neoplasm cells were weakly positive for CD8 (200 ×).

**Figure 3:**
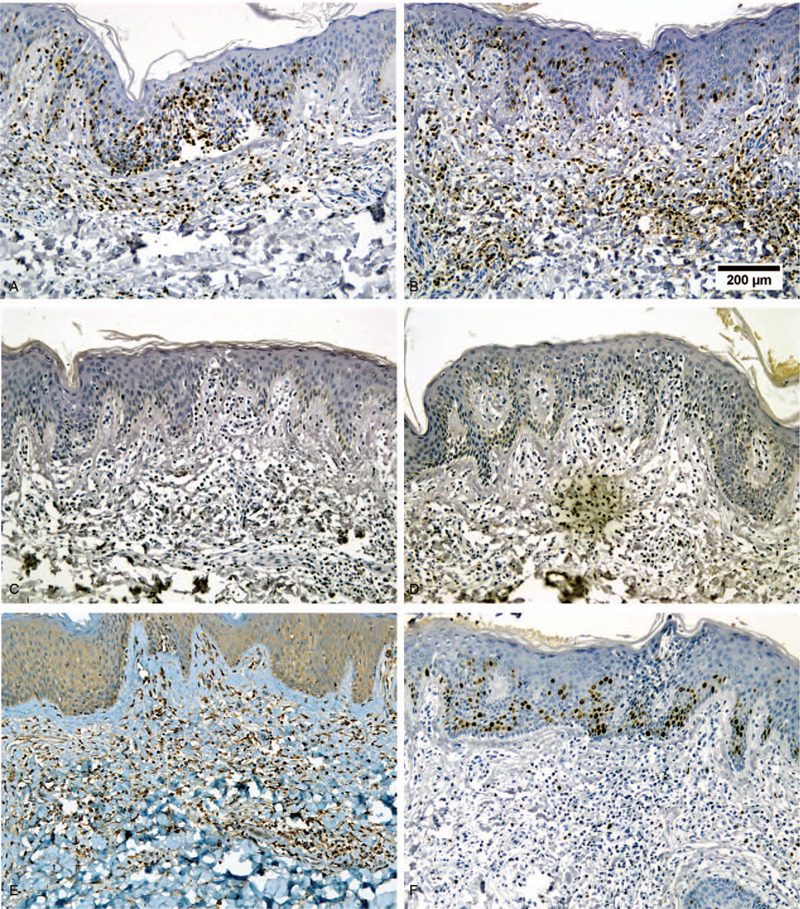
Immunohistochemical (IHC) assay. (A) Neoplasm cells were positive for CD3 (200 ×). (B) Neoplasm cells were positive for CD5 (200 ×). (C) Neoplasm cells were negative for CD20 (200 ×). (D) Neoplasm cells were negative for CD79a (200 ×). (E) Some cells were positive for TIA-1 (200 ×). (F) Neoplasm cells were positive for Ki-67 (<10%, 200 ×).

The dryness of the skin was alleviated, scales improved, and sweat was reduced by September 26, 2012. The patient's tongue was red with a thin and greasy coating, and pulse was fine and rapid. Microscopic examination revealed hyperkeratosis, parakeratosis, and mild acanthosis of the epidermis. Many atypical lymphocytes with medium-sized hyperchromatic nuclei were observed in the epidermis. Epidermotropism is characterized by lymphocyte colonization of the basal layer of the epidermis in a linear configuration. The superficial dermal infiltrate was band-like and scant (Fig. 2. A and B). Immunohistochemical staining showed that the atypical cells were positive for CD45RO, CD3, CD4, CD5, and lithocholic acid, weakly positive for CD7, CD8, and CD30, and negative for CD79a, CD56, CD68, CD20, granzyme-B, Perforin-T, and S-100. Some cells were positive for TIA-1. Ki-67 proliferative index was less 10% within the infiltrate. (Fig. 2. C, D and Fig. 3, Supplemental Digital Content (Figure S1)) In addition, gene rearrangement of T-cell receptor (TCR) γ was partially positive for V1J and weakly positive for V2J. TCR β showed V1J, V2J, and DJ were weakly positive, and TCR δ showed VJ, DD, and DJ were negative in clonal rearrangement of TCR genes. In summary, the final diagnosis was MF.

The previous misdiagnosis was corrected in time; however, the main composition of cooling blood and restrain Yang herbal medicine remained unchanged. The dose of drugs was adjusted appropriately, and the patient continued the use of the previously-described moisturizer on his skin.

The patient was followed up twice, on October 23, 2012 and on November 6, 2012. After taking the herbal medicine and using the moisturizer, the patient's dry skin and scales were alleviated and his sweating was significantly improved. The tongue was still red with the thin and greasy coating and the pulse fine and rapid; hence, the composition of herbal medicine was adjusted according to the clinical symptoms. After taking herbal medicine for 1 year, the symptoms improved greatly. The patient stopped taking oral herbal medicine in September 2013 in accordance with the advice of the doctor.

**Figure 4:**
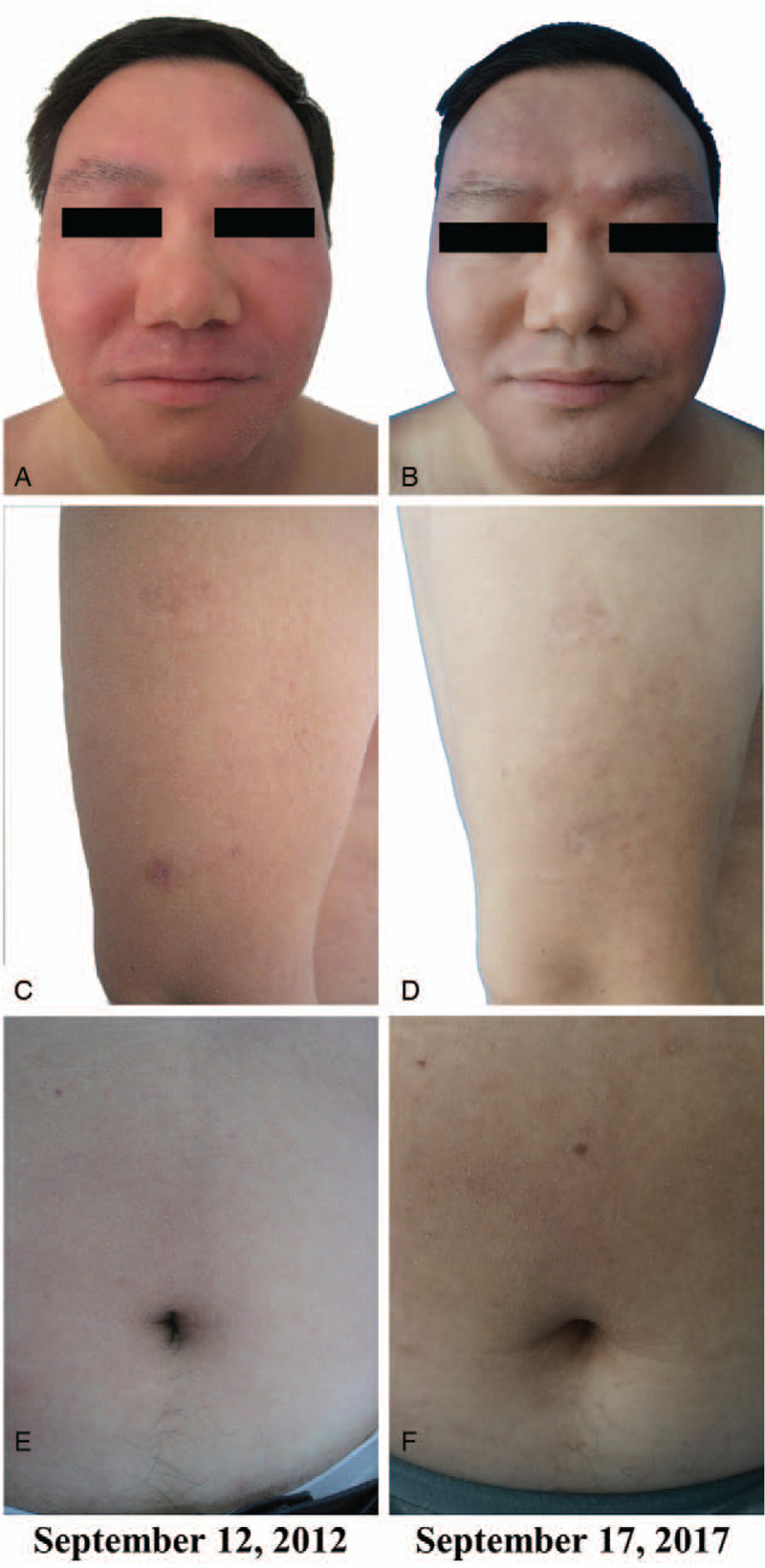
Clinical presentation of skin pre-treatment and post-treatment. (A, C, E) Appearance of the skin before treatment on September 12, 2012. (B, D, F) Appearance of the skin during the follow-up period on September 17, 2017. In the 5-year follow-up period, the dry and scaly skin and neck skin erythema were improved considerably compared to the pre-treatment condition.

**Figure 5:**
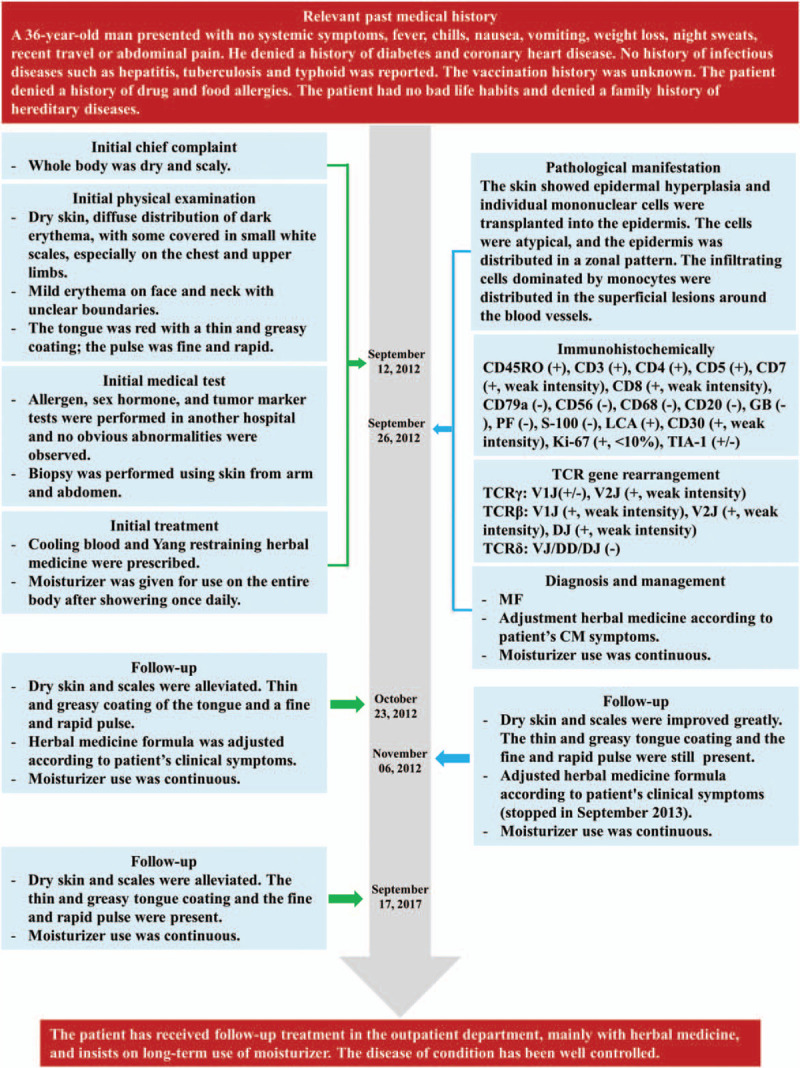
Timeline. Important dates, times, and detailed diagnosis process of the case in the treatment and follow-up period.

At presentation, the patient showed only dry and scaly skin, and the lesions did not involve internal organs or lymph nodes. Throughout the treatment and follow-up period, the patient showed good adherence and tolerance to the CM therapy. The patient's condition was well controlled and quality of life was improved. The patient has been regularly followed up in the clinic with no complaints of discomfort or adverse events occurring during the treatment. In 5-year follow up, the dry and scaly skin and erythema in neck were notably improved compared to the pre-treatment condition. The patient's skin lesions before and after treatment are shown in Figure 4. Full timeline of the diagnosis, medical treatment process, physical examination, clinical characteristics, and laboratory findings of the patient are shown in Figure 5.

## 3 Discussion

MF is the most common cutaneous T-cell lymphoma. MF presents a chronic progressive course. The diagnosis of MF in its patch or early plaque stage is difficult, as its clinical features resemble other benign dermatoses.^[16,17]^ The early stage of MF is easily confused with chronic dermatitis, psoriasis, vitiligo, and pigmented purpuric dermatitis, leading to delayed diagnosis and misdiagnosis.^[18]^ Therefore, positive histological markers of MF cells are of considerable value in the diagnosis of this disease.^[19]^ MF is mostly characterized by an infiltrate of T-helper memory lymphocytes expressing CD3, CD4, CD5, CD45RO; these cells are traditionally CD8 negative.^[20]^ CD4^+^ T cells participate in a variety of immune responses, which play vital roles in anti-tumor immunity and inflammatory responses. CD8^+^ T cells are key players in controlling infections and tumors.^[21,22]^ The prognosis of CD8^+^ MF is controversial. One study indicated that the prognosis of early MF is not influenced by phenotype,^[23]^ whereas others indicated that patients with CD8^+^ MF have a lower rate of disease progression, an indolent disease course, and a good prognosis.^[24,25]^ CD8^+^ MF shows special clinical features, such as hyperpigmentation and poikiloderma, which, when considering its slow progression, indicate a mild biological behavior.^[26]^ Although CD4/CD8 dual-positive MF is rare, it has been described. A literature search conducted using CD4/CD8 dual-positive MF as the key word resulted in the retrieval of 4 related case reports. Case characteristics of CD4/CD8 dual positive MF in the literature are summarized in Table 2A and Table 2B.^[7–10]^ The clinical manifestations of the 4 patients were different. Only 1 patient had lymph node, peripheral blood, and cardiac involvement, and was treated with chemotherapy. We have summarized the results of these reported cases in Table 2, including clinical characteristics, treatment, and immune phenotype in the hopes of providing further suggestions for the diagnosis and treatment of CD4/CD8 dual-positive cases.

**Table 2:**
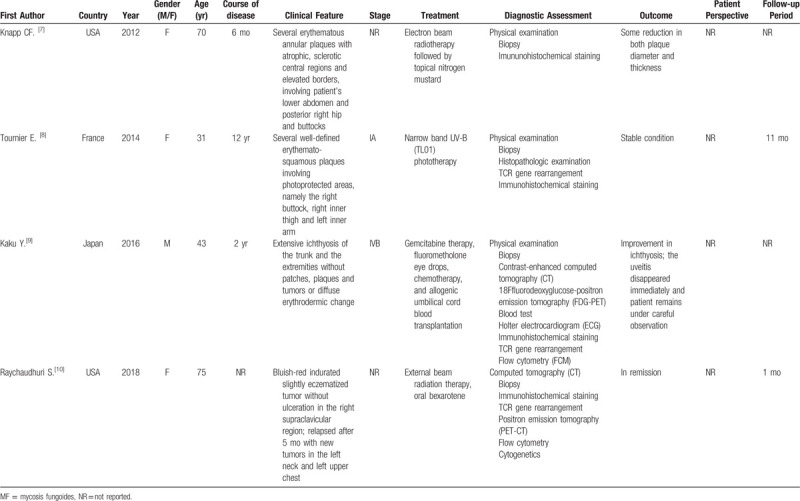
a. Summary of CD4/CD8 double-positive MF cases.

**Table 3:**
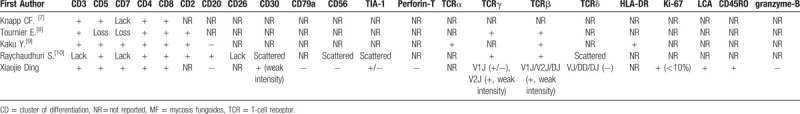
b. Immunophenotypic and molecular characteristics of patient lesions of MF CD4/CD8 dual-positive.

It is known that lymphoma cells are diffusely positive for CD3 and TCR γ, mostly positive for granzyme-B and TIA-1, variably positive for CD7, CD8, and CD30, and negative for CD4 and TCR β.^[27]^ In some cases, when CD7 is strongly positive, the diagnosis of lymphoma needs to be ruled out. Combined with the results of TCR gene rearrangement, the final diagnosis of MF was clear. The patient had red erythema on the face and neck but no obvious papule; mossy, ichthyosiform, poikiloderma; blisters and purpura-like rash, which are common and diverse manifestations of MF in the early stage. CM diagnosis as Yifeng sores, with blood heat and floating Yang as its basic pathogenesis. As the patient was in the early stages of MF and there was no involvement in other organs, we treated the patient with CM and moisturizer. The herbal medicine we gave to the patient cooled the blood and cleared heat, calming the mind. Moisturizer containing ceramide-linoleic acid can stimulate lipid production, epidermal differentiation, and aquaporin 3 expression, as well as restore epidermal permeability.^[15,28]^ The patient received follow-up treatment in the outpatient department, mainly with herbal medicine, and insisted on long-term use of moisturizer. The disease condition has been well controlled.

Although the disease progresses chronically, it inevitably develops into the tumor stage. Therefore, early diagnosis and early intervention to stabilize the condition and alleviate symptoms are crucial. In western medicine, the treatment of early lesions may include application of psoralens in association with UV-A irradiation, interferon α-2a, or retinoids. UV-B irradiation, topical application of chemotherapy agents and new active drugs are also used.^[26,29]^ In our case, herbal medicine was used to control the progression of the disease effectively. Efficacy of herbal medicine treatment may be related to the immune regulation effect of some special herbal medicines, but further studies are needed to confirm the mechanism of action of the medicine used for treatment. There are few reports on the management of MF by CM therapy, indicating a lack of experimental and clinical evidence. In the case we reported, the clinical symptoms of dryness and scales were relieved largely through the intervention of CM. We started with the pathogenesis of blood heat and floating Yang of the patient, and treated the syndrome accordingly. The patient showed high compliance in the process of receiving CM treatment, and no adverse reactions were observed after administration of the medicine. Overall, the tolerance of the patient using CM was better than traditional western medicine.

In this MF case, the immunophenotype involved co-expression of CD4 and CD8 without aggressive extracutaneous involvement. The patient only felt discomfort in the skin and the disease did not have a particularly serious impact on daily life. Overall, the disease progressed slowly and was well controlled in the years from onset to follow-up, which may be related to the positive expression of CD8. However, we cannot easily make an accurate clinical conclusion of a rare immunophenotype, which also puts forth new challenges and requirements for the diagnosis and treatment of diseases in the future. Diverging from previously reported cases, this case was treated with CM therapy. The key to CM treatment is to accurately identify the syndrome and treat the symptoms in combination with the patient's tongue coating, pulse, and symptoms. In terms of improving patient condition, CM may achieve unexpected results beyond our expectations. However, the efficacy, safety, and mechanisms of CM therapy in MF remain to be explored in further studies. Additionally, whether CM therapy or CM combined with western medicine therapy has better clinical efficacy for the treatment of MF needs to be confirmed clinically.

## Author contributions

**Conceptualization:** Xiaojie Ding, Xin Li.

**Data curation:** Jia Chen, Le Kuai.

**Formal analysis:** Meng Xing, Ying Luo.

**Funding acquisition:** Xin Li, Bin Li.

**Investigation:** Yi Ru, Ying Luo.

**Visualization:** Yue Luo, Mi Zhou.

**Writing –original draft:** Xiaojie Ding, Jia Chen.

**Writing –review & editing:** Xin Li, Bin Li.

## Supplementary Material

Supplemental Digital Content
