# Supplementary material for: CD4/CD8 dual-positive mycosis fungoides: A case report and literature review
Source: Medicine (Baltimore). 2020 Oct 16;99(42):e22786. doi: 10.1097/MD.0000000000022786 (PMC7571916; doi:10.1097/MD.0000000000022786)

**Supplementary** **Digital Content**

**Figure S1. Immunohistochemical (IHC) assay.** (a) Neoplasm cells were positive for LCA (200 ×). (b) Neoplasm cells were positive for CD45RO (200 ×). **(**c) Neoplasm cells were positive for CD7 (200 ×). (d) Neoplasm cells were weakly positive for CD30 (200 ×). (e) Neoplasm cells were negative for CD56 (200 ×). (f) Neoplasm cells were negative for CD68 (200 ×). (g) Neoplasm cells were negative for S-100 (200 ×). (h) Neoplasm cells were negative for Perforin-T (200 ×). (i) Neoplasm cells were negative for Granzyme-B (200 ×).


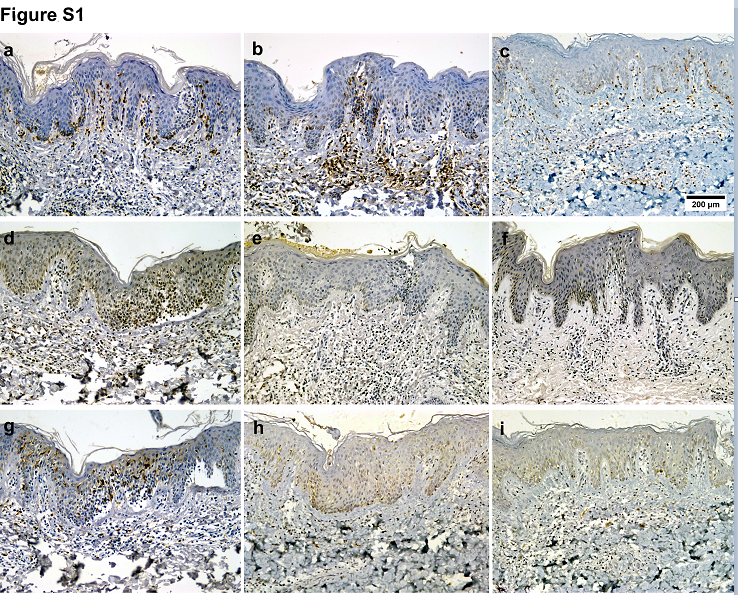

Supplement: Supplemental Digital Content [file medi-99-e22786-s001.doc]
